# Quantitative Synthetic Magnetic Resonance Imaging for Brain Metastases: A Feasibility Study

**DOI:** 10.3390/cancers14112651

**Published:** 2022-05-27

**Authors:** Amaresha Shridhar Konar, Akash Deelip Shah, Ramesh Paudyal, Maggie Fung, Suchandrima Banerjee, Abhay Dave, Vaios Hatzoglou, Amita Shukla-Dave

**Affiliations:** 1 Department of Medical Physics, Memorial Sloan Kettering Cancer Center, New York City, NY 10065, USA; konarsha@mskcc.org (A.S.K.); paudyalr@mskcc.org (R.P.); 2 Department of Radiology, Memorial Sloan Kettering Cancer Center, New York City, NY 10065, USA; shaha@mskcc.org (A.D.S.); hatzoglv@mskcc.org (V.H.); 3 General Electric Health Care, New York City, NY 10065, USA; maggie.fung@med.ge.com (M.F.); suchandrima.banerjee@ge.com (S.B.); 4 Touro College of Osteopathic Medicine, New York City, NY 10027, USA; adave3@student.touro.edu

**Keywords:** MAGnetic resonance image Compilation, normal-appearing brain tissue, brain metastases, MRI relaxometry

## Abstract

**Simple Summary:**

This preliminary study aims to characterize brain metastases (BM) using T1 and T2 maps generated from newer, rapid, synthetic MRI (MAGnetic resonance image Compilation; MAGiC) in a clinical setting. In addition, synthetic MR could provide contrast images analogous to standard T1- and T2-weighted images. The reproducibility and repeatability of this method have been previously established for brain imaging. This study reports and analyzes the quantitative T1 and T2 values for 11 BM patients (17 BM lesions) with a total of 82 regions of interest (ROIs) delineated by an experienced neuroradiologist. The initial results, which need to be further validated in a larger patient cohort, demonstrated the ability of T1 and T2 metric values to characterize BMs and normal-appearing brain tissues. The T1 and T2 metrics could be potential surrogate biomarkers for BM free water content (cellularity) and tumor morphology, respectively.

**Abstract:**

The present preliminary study aims to characterize brain metastases (BM) using T1 and T2 maps generated from newer, rapid, synthetic MRI (MAGnetic resonance image Compilation; MAGiC) in a clinical setting. We acquired synthetic MRI data from 11 BM patients on a 3T scanner. A multiple-dynamic multiple-echo (MDME) sequence was used for data acquisition and synthetic image reconstruction, including post-processing. MDME is a multi-contrast sequence that enables absolute quantification of physical tissue properties, including T1 and T2, independent of the scanner settings. In total, 82 regions of interest (ROIs) were analyzed, which were obtained from both normal-appearing brain tissue and BM lesions. The mean values obtained from the 48 normal-appearing brain tissue regions and 34 ROIs of BM lesions (T1 and T2) were analyzed using standard statistical methods. The mean T1 and T2 values were 1143 ms and 78 ms, respectively, for normal-appearing gray matter, 701 ms and 64 ms for white matter, and 4206 ms and 390 ms for cerebrospinal fluid. For untreated BMs, the mean T1 and T2 values were 1868 ms and 100 ms, respectively, and 2211 ms and 114 ms for the treated group. The quantitative T1 and T2 values generated from synthetic MRI can characterize BM and normal-appearing brain tissues.

## 1. Introduction

Magnetic resonance imaging (MRI) is one of the most widely employed imaging modalities for assessing brain tumors, owing to its noninvasive provision of high-resolution structural (T1- (spin–lattice) and T2- (spin–spin) weighted) images and functional (diffusion, perfusion, and metabolic) information [1]. Qualitative multiplanar T1 and T2 images (in particular post-Gadolinium T1-weighted) have been widely used for the anatomical location and morphological characteristics in brain tumors and metastases [2,3]. The varying signal intensities of T1 and T2 are based on the differences in the brain tissue’s intrinsic relaxation times associated with acquisition parameters (e.g., flip angle, echo time, and repetition time) and can be quantitatively mapped to measure pathophysiological changes [4,5]. The measurements of quantitative (q) relaxometry metrics may serve as surrogate biomarkers for free water content (cellularity) [6] and tumor morphology [7]. In clinical practice, standard quantitative T1 and T2 acquisition methods are time-consuming and not practically feasible in busy clinics [8,9,10,11,12]. Furthermore, the maps can be challenging to interpret due to low spatial resolution [13]. A single, time-efficient acquisition method that could simultaneously measure multiple tissue properties would account for these limitations and is therefore an unmet need in clinical brain imaging.

Rapid MRI acquisition methods have been developed to address the above technical limitations, showing promise in brain imaging [14,15,16]. Synthetic MRI is a newer, rapid method that simultaneously offers qualitative images and quantitative T1 and T2 maps in a clinically feasible timeframe [15,17,18]. The computational viability of data acquisition and processing to implement synthetic MRI was first tested for the brain [17]. The synthetic MR images were mathematically derived to display image contrast analogous to standard T1- and T2-weighted images [19]. The reproducibility and repeatability of this method have been vigorously tested and established for brain imaging [20]. A major vendor, General Electric Healthcare (GEHC, Waukesha, WI, USA), has developed synthetic MRI and termed the product MAGiC (MAGnetic resonance image Compilation), which is used in this study [21].

The quality and diagnostic accuracy of synthetic contrast-weighted images have been compared with standard images in the brain [21,22]. Tanenbaum et al. performed a prospective trial on 109 subjects with neuroimaging indications, in which 1526 MR images were read by seven blinded neuroradiologists to compare synthetic qualitative images with standard brain MRI [21]. They concluded that the synthetic MR imaging quality was similar to that of standard proton density (PD), short tau inversion recovery (STIR), and T1- and T2-weighted contrast views across neurologic conditions. While artifacts were more common in synthetic T2 fluid attenuated inversion recovery (FLAIR), these were readily recognizable, did not mimic pathology, and only necessitated additional standard T2 FLAIR to confirm diagnosis [21]. This study extensively focused on qualitative image analysis, not quantitative relaxometry maps [21]. Hagiwara et al. studied ten patients with a combined total of 167 brain metastases (BM) lesions and reported that the T1 inversion recovery (IR) qualitative images generated by the synthetic MRI method in BM patients created better contrast than synthetic T1-weighted or standard T1 IR imaging [23]. Furthermore, the detection of brain metastases was comparable among these qualitative images [23].

Recently, Blystad et al. demonstrated the promise of quantitative synthetic MRI to assess nonvisible peritumoral contrast enhancement in the peritumoral edema of malignant gliomas via the change (Δ) in R_1_ (R_1_ = 1/T_1_) maps [24]. In accordance, Müller et al. previously showed that standard quantitative T1 mapping could discover a subtle enhancement in glioblastoma patients, which is not visually detectable with T1-weighted subtraction images [25]. These findings have clinical implications, as an early reduction in the enhancing region under therapy predicts a favorable therapy response [24,25].

We will focus on BM as it is the most common intracranial tumor in adults. Lung cancer, breast cancer, and melanoma are the most common primary tumor sites [26]. The incidence of BM has risen due to the increased rates of diagnosis and increased survival of cancer patients [27,28]. Multiparametric MRI, including quantitative relaxometry metrics, may further improve detection and treatment selection [29,30,31]. A recently published study measured the magnetic resonance relaxation time using a multi-dynamic multi-echo (MDME) sequence at three time points (1, 10, and 20 min) after contrast injection on seven BM patients and showed that these measurements were time-dependent [32]. The present preliminary study aims to investigate the value of T1 and T2 metric values derived from the newer, rapid synthetic MRI method (MAGiC) and assess their ability to characterize untreated and treated BM, as well as normal-appearing brain tissue.

## 2. Materials and Methods

### 2.1. Phantom Selection

An MRI system phantom procured from CaliberMRI (Boulder, CO, USA) was co-developed by International Society for Magnetic Resonance in Medicine (ISMRM)/National Institute of Standards and Technology (NIST) to provide a standard phantom to perform qMRI studies, including and not limited to T1 and T2 relaxation times [33]. MRI system phantom consists of 14 vials in each T1 and T2 array with appropriate chemical composition to provide precise T1 and T2 values, including the range of T1 and T2 values generally seen in gray and white matter of the brain. The Nuclear Magnetic Resonance (NMR)-based reference T1 and T2 relaxation times were provided by NIST. This phantom has SI-traceable components and was monitored for its stability and accuracy [34].

### 2.2. Patient Selection

The Institutional Review Board (IRB) approved this Health Insurance Portability and Accountability Act (HIPAA)-compliant prospective study for participating patients. Written informed consent was obtained from all eligible 21 patients with brain tumors. This study focuses on 14 patients who had BM. In final analysis, we investigated the data acquired from 11 patients who had a total of 17 evaluable BM lesions. We excluded three patients due to small lesion size <0.5 mm. There is a subset of patient overlap with our companion paper on Brain Magnetic Resonance Fingerprinting (MRF) in this same journal issue. MRF and synthetic MRI are two different MRI relaxometry techniques. There is no overlap in image acquisition and data analysis methods between these two studies.

The median age for these 11 BM patients was 52 years (range, 25–61 years; five male and six female). All BM patients were enrolled between June 2019 and August 2021. The inclusion criteria were age ≥ 18 years, and clinical or radiological diagnosis of BM. Patient characteristics are illustrated in Table 1.

Patients with BM underwent standard MRI, including MAGiC sequence, irrespective of the treatment group (untreated (*n* = 3) or treated (*n* = 8)). This study focuses on testing the MAGiC sequence in BM patients. They were scanned at a single time point, not longitudinally. The therapy regimens for eight BM patients who underwent treatment were stereotactic radiosurgery (SRS) *n* = 3, focal radiation therapy (RT) *n* = 2, and whole-brain RT *n* = 3.

### 2.3. MRI Data Acquisition

ISMRM/NIST system phantom was scanned on GE (General Electric Healthcare, Waukesha, WI, USA) MRI system (Discovery 3.0 T MR750w) using an eight-channel brain array coil. The Gold Standard (GS) T1 measurements from the T1 arrays were acquired by the IR spin echo method with specific acquisition parameters as follows: inversion time (TI) = 50, 75, 100, 125, 150, 250, 500, 1000, 2000, 3000 ms; repetition time (TR) = 4500 ms; echo time (TE) = 7.34 ms; acquisition matrix 128 × 128; matrix reconstructed to 256 × 256; field of view (FOV) = 25 cm; slice thickness = 5 mm. The scan time for each TI measurement was approximately four minutes and the total scan time for GS T1 acquisition was around 40 min. The GS T2 measurements from the T2 array were obtained using a multiple single-echo spin echo method with the following acquisition parameters: TEs = 9, 12, 15, 20, 25, 30, 40, 45, 50, 60, 75, 80, 100, 120, 160 ms; TR = 5000 ms; acquisition matrix 128 × 128; matrix reconstructed to 256 × 256; FOV = 25 cm; slice thickness = 5 mm. The scan time of each TE measurement was approximately 21: 30 (min: sec); the total scan time was approximately five hours. The nonlinear least-squares curve fitting was performed in MATLAB (The MathWorks. Inc., Natick, MA, USA). MAGiC phantom data were acquired in a coronal plane with an FOV of 25 cm. Remaining acquisition parameters used for the MAGiC phantom data acquisition were the same as the patient data acquisition detailed below.

All patient MRI examinations were performed on a GE MRI system (Discovery 3.0 T MR750w) using a 16-channel head and neck coil for brain imaging. Images were prospectively acquired using a fixed set of scanning parameters closely approximating the current standard-of-care for the brain. The standard clinical MR acquisition parameters for brain imaging was multiplanar (axial, coronal, and sagittal). In this study, we used conventional axial T1-weighted (w) (both pre- and post-contrast images), T2w fat-suppressed, and T2w FLAIR images were acquired pre-contrast with slice thickness (ST) of 3 mm and FOV of 20–24 cm. The acquisition parameters were as follows: T1w imaging: TR = 2000 ms, TI = 1101 ms; TE = 25 ms; number of averages (NA) = 1; acquisition matrix 320 × 224; matrix reconstructed to 320 × 256; scan time ~2.43 min. T2w imaging with fat-suppressed fast spin echo: TR = 4796 ms; TE = 121 ms; NA = 1; acquisition matrix 256 × 256; matrix reconstructed to 320 × 256; scan time ~2.27 min. FLAIR imaging: TR = 9946 ms; TE = 127 ms; TI = 2375 ms; NA = 1, acquisition matrix 256 × 192; matrix reconstructed to 256 × 256, scan time ~5.13 min.

MAGiC brain data were acquired prior to contrast agent injection using multiple-dynamic multiple-echo (MDME) sequence, followed by synthetic image reconstruction and post-processing. MDME is a multi-contrast sequence and uses a multi-echo acquisition that enables absolute quantification of physical tissue properties, including T1, T2, and proton density (PD) independent of scanner settings. It uses multiple inversion times (TIs) to measure T1 relaxation. MDME parameters acquired in one scan are used in synthetic imaging to calculate pixel intensity and produce an appearance similar to standard MR images with modifiable TE, TR, and TI [35]. This provides quantitative (T1, T2, and PD maps) and qualitative images (T1, T2, T1 FLAIR, T2 FLAIR, PD, and STIR contrast images). Two-dimensional axial MAGiC brain data were acquired with four automatically calculated saturation delays, two echo times (TE) (23.4 ms and effective TE of 93.8 ms), and an auto TR of 4000 ms (ranging between 4000 ms–15,000 ms). The other parameters were FA = 90°; Echo Train Length (ETL) = 12; ST = 5 mm; FOV = 25 cm; acquisition matrix 320 × 256; matrix reconstructed to 256 × 256. Total scan time was approximately 6 min.

### 2.4. MRI Data Post-Processing

MAGiC is a combined package of image acquisition and a post-processing. MAGiC post-processing generates parametric maps of both the T1 and T2 relaxation times, as well as PD, using the data obtained by running the MDME sequence. Contrast-weighted T1, T2, T1 FLAIR, T2 FLAIR, STIR, phase-sensitive inversion recovery (PSIR), and double inversion recovery (DIR) images can be synthesized using the quantitative maps for any desired combination of TE, TR, and TI. The contrast-weighted image settings can be defined on the post-process screen or the MAGiC session application. This study focuses on the estimation of the quantitative T1 and T2 parametric maps for BM patients.

### 2.5. Regions of Interest Delineation

Regions of interest (ROI) were delineated by a neuroradiologist with over seven years of experience. To determine the extent of the tumor, anatomical T2w and contrast-enhanced T1w images were used. ROIs were drawn, excluding cystic and necrotic regions. Based on the literature, we modified the lesions’ size threshold for ROI delineation to ≥5 mm [36]. The size of the BM lesions ranged between 5 mm and 26 mm in this study. MAGiC-derived mean T1 and T2 metric values were obtained from the selected ROIs for normal and tumor tissues.

The neuroradiologist delineated 17 ROIs on the central slice of the evaluable BM lesions. ROIs were also delineated in normal-appearing gray matter (GM), white matter (WM), and cerebrospinal fluid (CSF) for the eight patients (three untreated; five treated with SRS or focal RT). We did not assess the GM, WM, and CSF for the three patients who received whole-brain RT as there can be microscopic changes in the normal-appearing tissues after radiation exposure. MAGiC simultaneously generates T1 and T2 maps. Hence, ROIs were drawn on the T1 map and the same ROIs were used for T2 maps to obtain the mean value. In total, 82 ROIs were analyzed for all patients in this study.

### 2.6. Statistical Analysis

For the T1 and T2 values obtained using the phantom, the relative percentage difference between the method (phantom vendor provided (VP), GS acquisition, and the MAGiC) was calculated. The univariate analysis was performed on the mean metric value extracted from the ROIs using the Wilcoxon Rank Sum test (WRST) to find the difference between the untreated and treated groups. WRST is also called the Wilcoxon Mann–Whitney test and the Mann–Whitney U test to compare two independent samples. The T1 and T2 values from both untreated and treated groups were compared with the healthy-appearing brain tissues. In addition, T1 and T2 values (mean, median, and range) were reported from healthy-appearing GM, WM, and CSF. For WRST, the significance level was set at *p* ≤ 0.05.

## 3. Results

Table 2 reports the T1 and T2 values, as well as the relative percentage difference, between the three methods (VP, GS, and MAGiC). The percentage difference between the methods for T1 values showed a maximum of 6.6% between VP and GS, 16% between VP and MAGiC, and 23.9% between GS and MAGiC. Similarly, the percentage difference between the methods for T2 values showed a maximum of 16.8% between VP and GS, 18.4% between VP and MAGiC, and 30.5% between GS and MAGiC.

**Figure 1:**
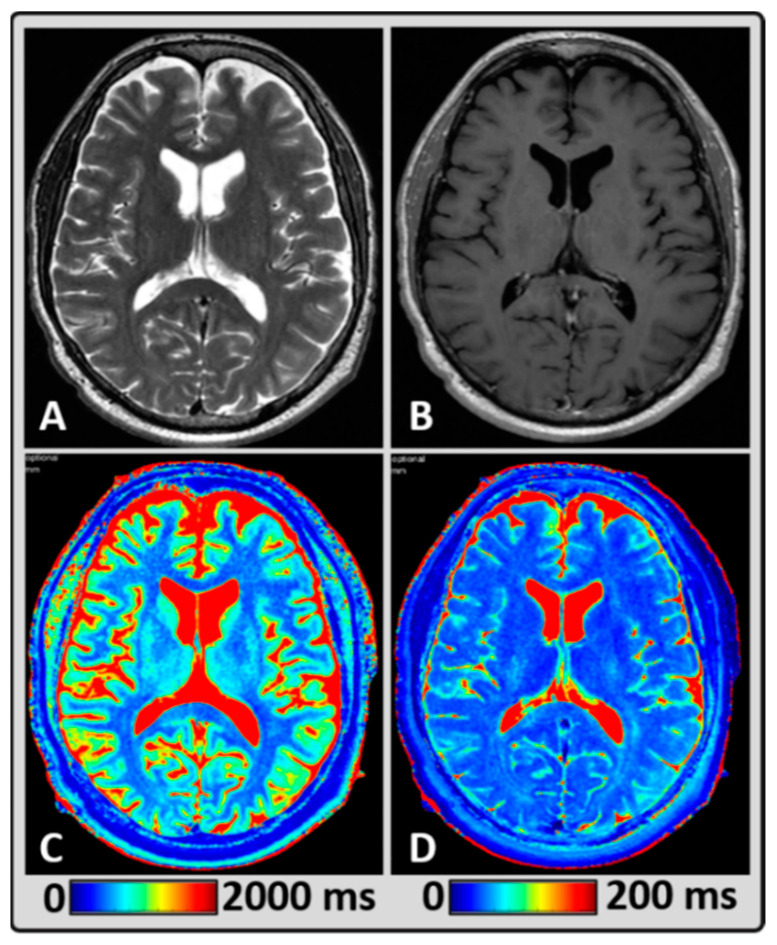
Representative MRI data from a 58-year-old male BM patient treated with focal radiation therapy (RT), showing normal-appearing brain tissue. (**A**,**B**) standard T2w and post-contrast T1w images depicting the anatomical structures, (**C**,**D**) T1 and T2 maps were estimated using MAGnetic resonance image Compilation (MAGiC) for a single slice exhibiting gray matter (GM), white matter (WM), and cerebrospinal fluid (CSF).

Figure 1 shows qualitative (standard clinically acquired T2w and post-contrast T1w images) and quantitative (T1 and T2 maps generated from MAGiC for normal-appearing GM, WM, and CSF) MRI data from a representative BM patient treated with focal RT. The mean T1 values for normal-appearing GM, WM, and CSF regions were 1098 ms, 767 ms, and 4289 ms, respectively. The mean T2 values were 75 ms, 65 ms, and 394 ms, respectively.

**Figure 2:**
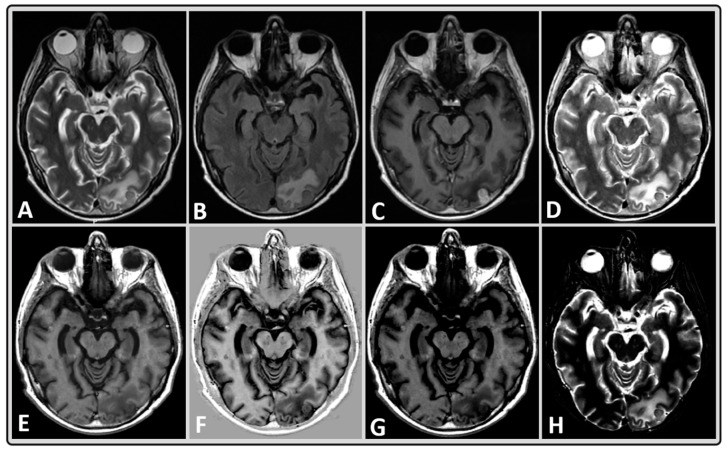
Representative MRI data of a 40-year-old male BM patient treated with whole-brain RT using standard and synthetically-generated (MAGiC) images. (**A**–**C**) T2w, T2w fluid attenuated inversion recovery (FLAIR) and T1w post-contrast images, respectively, from standard clinical imaging. (**D**–**H**) T2w, T1w, phase-sensitive inversion recovery (PSIR), T1w FLAIR, and short tau inversion recovery (STIR) images, respectively, from MAGiC.

Figure 2 shows representative qualitative, multi-contrast-weighted images that were synthesized at the fixed combination of TE, TR, and TI from a BM patient treated with whole-brain RT. This figure illustrates that multiple diagnostic images can be obtained in a rapid, single MRI acquisition in a clinically feasible time.

**Figure 3:**
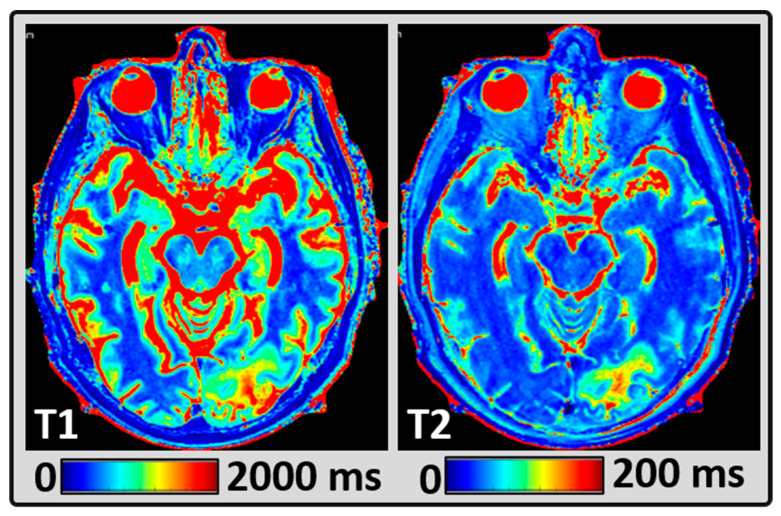
Representative MRI data from the same patient as Figure 2, showing quantitative images. (A and B) synthetically-generated T1 and T2 maps, respectively, from MAGiC.

Figure 3 shows the representative quantitative T1 and T2 maps from the same patient as Figure 2. The mean T1 and T2 values measured within the BM lesions were 1933 ms and 96 ms, respectively.

**Figure 4:**
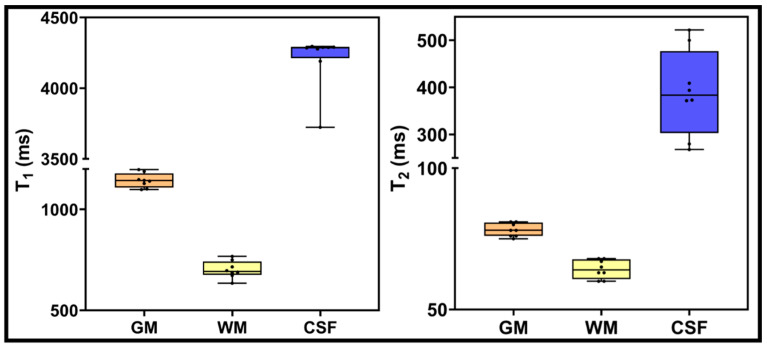
T1 and T2 values for normal-appearing GM, WM, and CSF using T1 and T2 maps generated from MAGiC methods. Boxes represent the interquartile range; whiskers represent the range of all values; the horizontal line within the box is the median value.

The box plot in Figure 4 exhibits T1 and T2 values obtained from normal-appearing GM, WM, and CSF regions using MAGiC-generated T1 and T2 maps. GM mean values for T1 and T2 were 1143 ms and 78 ms, respectively. Mean T1 and T2 values for WM were 701 ms and 64 ms, respectively. Mean T1 and T2 values for CSF regions were 4206 ms and 390 ms, respectively.

Table 3 shows the mean and range for T1 and T2 values estimated from MAGiC for BM lesions (untreated *n* = 3; treated *n* = 8). Mean T1 and T2 values measured within BM lesions for the untreated group were 1868 ms and 100 ms, respectively. Similarly, the treated group’s mean T1 and T2 values were 2211 ms and 114 ms, respectively. In comparison, the WRST showed no significant difference for the T1 and T2 values from BM lesions between the treated and untreated groups (*p* > 0.05). However, WRST performed for the mean T1 and T2 values between the BM and normal-appearing WM showed significant differences for both the untreated and treated groups (*p* < 0.05).

**Figure 5:**
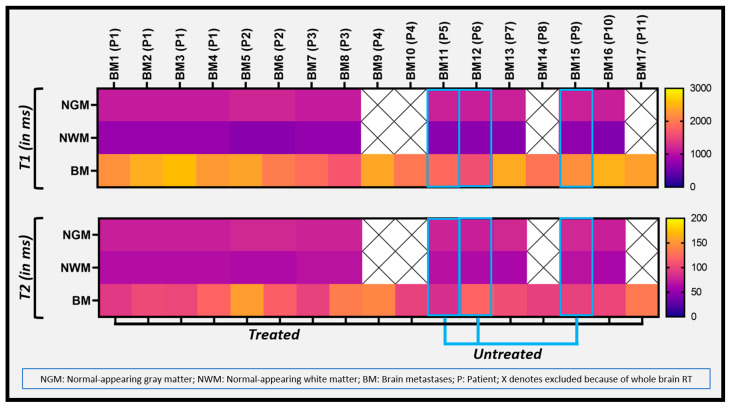
The heat map exhibits the T1 and T2 metric values of all the BM lesions and normal-appearing GM and WM.

Figure 5 depicts a heat map of the range of T1 and T2 values from all the BM lesions, exhibiting a clear difference between BM and normal-appearing brain tissue (GM and WM). All 17 metastases from 11 patients were analyzed separately (patients were coded as “P” and metastatic lesions as “BM”) and four patients had more than one metastasis.

## 4. Discussion

Quantitative MRI using relaxometry reveals fundamental information and absolute numerical values of tissue [37]. However, the long acquisition times associated with quantitative T1 and T2 measurements limit its clinical applications [8,9,10,11,12]. Reducing acquisition scan times can facilitate diagnosis, staging, and treatment selection [38]. Riederer et al. illustrated that synthesized MR images can be generated using arbitrary pulse-sequence parameters [15,39]. Feasibility studies have previously reported that synthetic MRI-generated T1 and T2 maps were diagnostically acceptable in the normal brain [21,40,41,42]. The utility of this method has been further tested for extracranial organs, including the prostate, breast, and rectum [43,44,45,46]. The present study tested the robustness of the MAGiC method for the quantitative measurement of T1 and T2 values on a phantom and patients with BM. The accurate measurement of T1 and T2 values is critical in qMRI applications to probe tissue physiology. Measured Gold Standard (GS) T1 and T2 values would provide further evidence of the robustness of relaxometry mapping performed using the MAGiC method. GS acquisition is time-consuming and practically challenging to perform on patients. Therefore, we performed an additional experiment using the ISMRM/NIST MRI system phantom. We evaluated the robustness of MAGiC T1 and T2 measurements on the phantom by comparing vendor-provided data and data acquired with the GS method. We considered the T1 and T2 values from only seven vials that mimic brain tissue [9,47].

In our preliminary experience, synthetic MRI-generated T1 and T2 maps exhibited tumor heterogeneity for the first time and the metric values were able to differentiate between normal-appearing tissue and BM lesions. Using a 3T MRI and standard T1 and T2 mapping methods, Wansapura et al. reported mean T1 values of 1331 ms and 832 ms, and mean T2 values of 110 ms and 80 ms for normal-appearing GM and WM, respectively [9]. Our quantitative synthetic MRI results reported similar T1 and T2 values for normal-appearing GM and WM as in their published study [9].

Intrinsic T1 and T2 image contrast depends on the composition of tissues, including tissue water content, iron concentration, and the relative proportion of various macromolecules [48]. The quantification of T1 and T2 provides surrogate metrics of the underlying pathophysiology with tissue measurement of free water content and vascular morphology [12]. Quantitative T1 and T2 values may reflect an alteration in tissue composition and are surrogate biomarkers for tumor water content and morphology. These differences can explain variations in macromolecular tissue compositions. By adjusting the MR scan parameters for tissues, we can improve our ability to capture these changes. Quantifying relaxation times and proton density by the multi-echo acquisition of a saturation-recovery using a turbo spin echo readout sequence on a 1.5T MRI, Warntjes et al. reported T1 and T2 values of 561 ms and 73 ms, respectively, in the frontal white matter of the adult brain [35]. In the present study, the newer, rapid MAGiC method synthetically generated multiple contrast-weighted images at a fixed combination of TE, TR, and TI for all BM patients. As Tanenbaum et al. have exhaustively demonstrated the similar image quality of multiple synthetically (MAGiC) generated images to that of diagnostic MRI across neurologic conditions [21], we did not report here on qualitative diagnostic comparisons for brain imaging. Tanenbaum et al. also pointed out that, though artifacts were more common in synthetic T2 FLAIR, these were readily recognizable and did not mimic pathology, but they could necessitate additional standard T2 FLAIR to confirm diagnosis [21]. It has also been previously reported that BM detection was comparable among qualitative synthetic and standard MR images [21].

The implementation of advanced quantitative imaging techniques requires significant time, technical, and cost resources. Therefore, the quantitative characterization of tissue properties eludes effective clinical utility in day-to-day practice. Synthetic MRI-generated multiple contrast images, including qualitative and quantitative T1 and T2 mapping, represent an alternative solution for clinical diagnostic imaging due to their rapid acquisition and data quantification. In addition, these newly developed methods hold promise for the identification of subtle enhancements in the peritumoral area that are not visible on standard imaging. These findings could have clinical implications as the early reduction in enhancing regions under therapy predicts a favorable therapy response [24,25].

The focus of this study was to test the ability of quantitative T1 and T2 values to characterize BM lesions and normal-appearing brain tissues. This study had several limitations. The sample size was small for both treated and untreated groups. The results, therefore, need to be validated in studies with a larger BM patient cohort. The slightly higher T1 relaxation times observed for untreated and treated BMs could be due in part to pathophysiological variations caused by differences in the primary tumor sites. This needs further investigation. The measured T2 value for CSF using MAGiC was underestimated, which may be attributed to factors such as CSF motion, eddy current effects, inadequate sampling of the relaxation curve, partial volume, and therefore warrants further investigation. The role of both synthetic qualitative and quantitative images in the diagnostic workup requires multi-center testing before incorporation into rapid brain imaging protocols for clinical use. Future studies may also evaluate synthetically-generated multiple contrast images using MAGiC for BM patients.

## 5. Conclusions

In conclusion, the present preliminary results demonstrate that quantitative T1 and T2 values from synthetic MRI can differentiate between BM and normal-appearing brain tissues. Further evaluation in a larger patient cohort is required to characterize BM’s pathophysiological features and to assess the longitudinal treatment response.

## Figures and Tables

**Table 1:** Patient characteristics.

Characteristics	Value
Total patients	11
Total number of BM lesions	17
Demographics	
Median age (y)	52
Age range (y)	25–61
Male/Female	5/6
Location of primary tumor	
Lung	5
Colon	1
Melanoma	2
Other	3
Untreated/Treated	3/8

**Table 2:** T1 and T2 values from three different methods.

**Vial #**	**T1 (in ms)**			**Percent of Difference (in %)**		
	**VP**	**GS**	**MAGiC**	**VP and GS**	**VP and MAGiC**	**GS and MAGiC**
1	1838	1779.7	1719	3.2	6.5	3.4
2	1398	1350.9	1179	3.4	15.7	12.7
3	998.3	957.7	852	4.1	14.7	11
4	725.8	678.2	622	6.6	14.3	8.3
5	509	483	453	5.1	11	6.2
6	367	345.9	327	5.7	10.9	5.5
7	258.7	242.1	300	6.4	16	23.9
**Vial #**	**T2 (in ms)**			**Percent of Difference (in %)**		
	**VP**	**GS**	**MAGiC**	**VP and GS**	**VP and MAGiC**	**GS and MAGiC**
1	645.8	537.4	591	16.8	8.5	10
2	423.6	357.4	414	15.6	2.3	15.8
3	286	245.9	287	14	0.3	16.7
4	184.8	162.6	186	12	0.6	14.4
5	134.1	118.3	141	11.8	5.1	19.2
6	94.4	81.6	103	13.6	9.1	26.2
7	62.5	56.7	74	9.3	18.4	30.5

VP: Phantom Vendor Provided; GS: Gold Standard; MAGiC: MAGnetic resonance image Compilation.

**Table 3:** T1 and T2 values in patients from the untreated and treated groups.

Relaxometry Values		Untreated BM	Treated BM
**T1** **(ms)**	Median (min, max)	1845 (1583,2177)	2311 (1654, 2558)
	Mean ± SD	1868 ± 298	2211 ± 269
**T2** **(ms)**	Median (min, max)	97 (85, 119)	104 (92, 154)
	Mean ± SD	100 ± 17	114 ± 20

## Data Availability

The data presented in this study will be provided upon reasonable request.
